# Acute on Chronic Neuromuscular Respiratory Failure in the Intensive Care Unit: Optimization of Triage, Ventilation Modes, and Extubation

**DOI:** 10.7759/cureus.16297

**Published:** 2021-07-10

**Authors:** Nick M Murray, Richard J Reimer, Michelle Cao

**Affiliations:** 1 Neurology, Stanford University School of Medicine, Palo Alto, USA; 2 Pulmonary and Critical Care, Stanford University School of Medicine, Palo Alto, USA

**Keywords:** neuromuscular disease, amyotrophic lateral sclerosis, acute respiratory failure, noninvasive ventilation, mechanical ventilation, airway clearance, extubation, quality and standardization, critical care

## Abstract

Critical care management of acute respiratory failure in patients with neuromuscular disease (NMD) such as amyotrophic lateral sclerosis (ALS) is not standardized and is challenging for many critical care specialists. Progressive hypercapnic respiratory failure and ineffective airway clearance are key issues in this patient population. Often at the time of hospital presentation, patients are already supported by home mechanical ventilatory support with noninvasive ventilation (NIV) and an airway clearance regimen. Prognosis is poor once a patient develops acute respiratory failure requiring intubation and invasive mechanical ventilatory support, commonly leading to tracheostomy or palliative-focused care.

We focus on this understudied group of patients with ALS without tracheostomy and incorporate existing data to propose a technical approach to the triage and management of acute respiratory failure, primarily for those who require intubation and mechanical ventilatory support for reversible causes, and also for progression of end-stage disease. Optimizing management in this setting improves both quality and quantity of life.

Neuromuscular patients with acute respiratory failure require protocolized and personalized triage and treatment. Here, we describe the technical methods used at our single institution. The triage phase incorporates comprehensive evaluation for new etiologies of hypoxia and hypercapnia, which are not initially presumed to be secondary to progression or end-stage neuromuscular respiratory failure. In select patients, this may involve intubation or advanced adjustments of NIV machines. Next, once the acute etiology(s) is identified and treated, the focus shifts: training and use of mechanical airway clearance to optimize pulmonary function, facilitation of NIV wean or successful extubation to NIV, and transition to a stable regimen for home ventilation. The comprehensive protocol described here incorporates multi-institutional approaches and effectively optimizes acute respiratory failure in patients with neuromuscular pulmonary disease.

## Introduction

Acute respiratory failure in chronic neuromuscular disease (NMD)

Respiratory failure is often an expected course and outcome in patients with NMD. In this technical report, we focus on patients with chronic respiratory failure presenting with acute respiratory decompensation. This patient cohort includes those with amyotrophic lateral sclerosis (ALS), spinal muscular atrophy (SMA), muscular dystrophies, and myopathies. There is limited evidence for guidance on the management of these patients due to the lack of randomized clinical trial data to inform best practices. Additionally, the severe progressive pathophysiology of respiratory failure affecting patients chronically before hospital presentation deviates from standard respiratory failure protocols and creates challenges to optimizing care in the acute setting.

Patients with progressive respiratory insufficiency secondary to NMD are often initiated on noninvasive ventilation (NIV) in the home setting. The most impressive set of data on NIV in neuromuscular respiratory failure was demonstrated in patients with Duchenne muscular dystrophy; NIV reduces hospitalization rates, prolongs survival, and has become the first line and standard of care for patients with NMD, especially those with SMA and ALS [1,2]. In studies of ALS patients with respiratory insufficiency, NIV prolonged median survival from 18 days to 298 days, improved quality of life, and the effect was more pronounced in patients with normal to mild bulbar weakness [3-5].

NIV has undergone significant advances in technology and carries major benefits for patients: lower cost compared to invasive mechanical ventilatory support with tracheostomy, fewer respiratory infections, increased comfort, and less burden on caregivers [6]. Therefore, avoiding or even just delaying tracheostomy is a common goal for patients, caregivers, and healthcare providers. 

Despite home use of NIV, patients with NMD often present to the hospital with acute respiratory compromise, however, this does not mean that there is a failure of outpatient NIV. Treatment often requires acute adjustment of the NIV strategy or intubation and mechanical ventilation. Intubation should be the default option if uncertainty exists as it safely stabilizes the patient and allows time to determine best next steps of therapy. Below, we describe our approach for patients with NMD who develop acute on chronic respiratory failure: intensive care unit (ICU) triage, ventilatory management, airway clearance approach, and an NMD-specific extubation protocol.

## Technical report

Standardized ICU Triage

Screening for Etiologies of Acute Respiratory Failure

**Figure 1:**
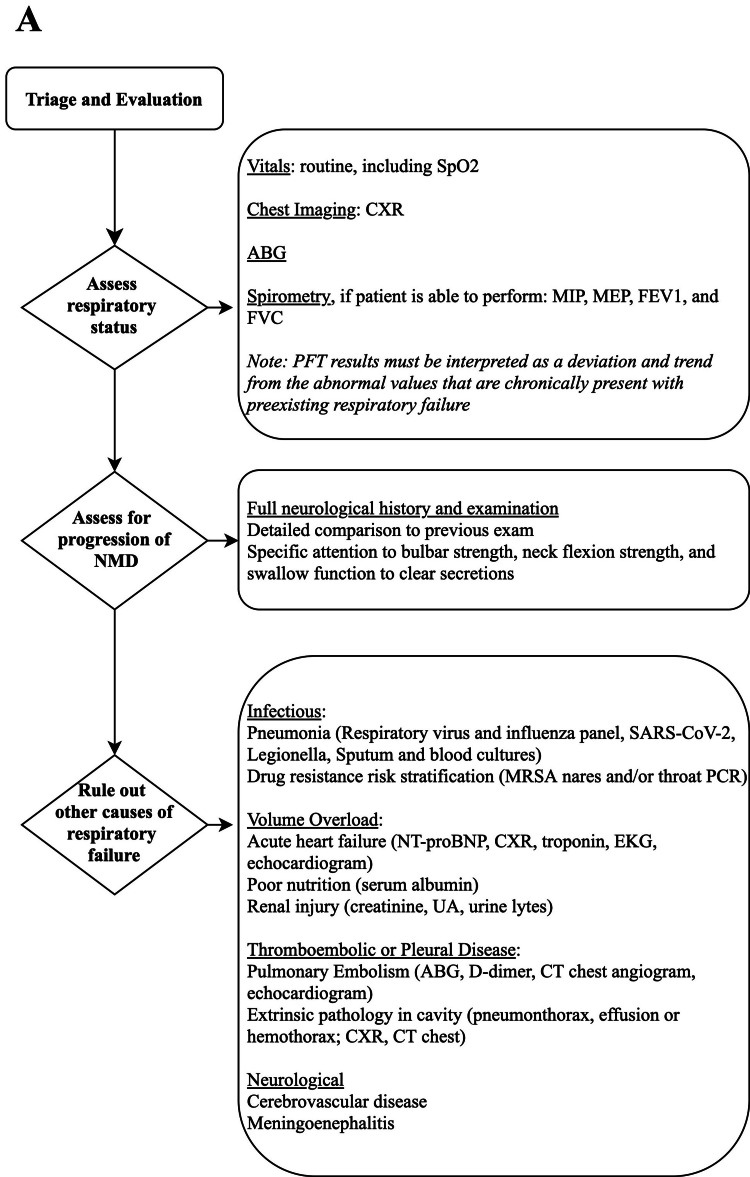
The critical care triage and evaluation of patients with acute on chronic neuromuscular failure A. Triage and evaluation of stabilizing respiratory function, assessment for progression of neuromuscular disease (NMD) as a cause of respiratory distress, and evaluation for other causes of respiratory failure. SpO2: pulse oximetry; CXR: chest X-ray; MIP: maximal inspiratory force; MEP: maximal expiratory force; FEV1: forced expiratory volume in 1 second; FVC: forced vital capacity; PFT: pulmonary function test; SARS-CoV-2: severe acute respiratory syndrome coronavirus 2; MRSA: methicillin-resistant staphylococcus aureus; NT-proBNP: N-terminal prohormone of brain natriuretic peptide; ABG: arterial blood gas; UA: urinalysis.

Acute on chronic NMD respiratory failure can present with hypoxemia and/or hypoventilation. A number of factors outside of NMD itself can cause acute respiratory compromise. Common causes include aspiration, pulmonary infections, thromboembolic events, excessive respiratory secretions, and mucus plugging. In those with cardiomyopathy, there is also a risk of acute heart failure. As some of these etiologies are reversible, patients should be screened with a comprehensive battery of tests (Figure 1). ICU admission is recommended due to potential for rapid deterioration in a patient with limited pulmonary reserve. In some situations, a step-down or intermediate care unit may be an acceptable level of care for more stable patients.

Decision to use Noninvasive versus Invasive Mechanical Ventilation

**Figure 2:**
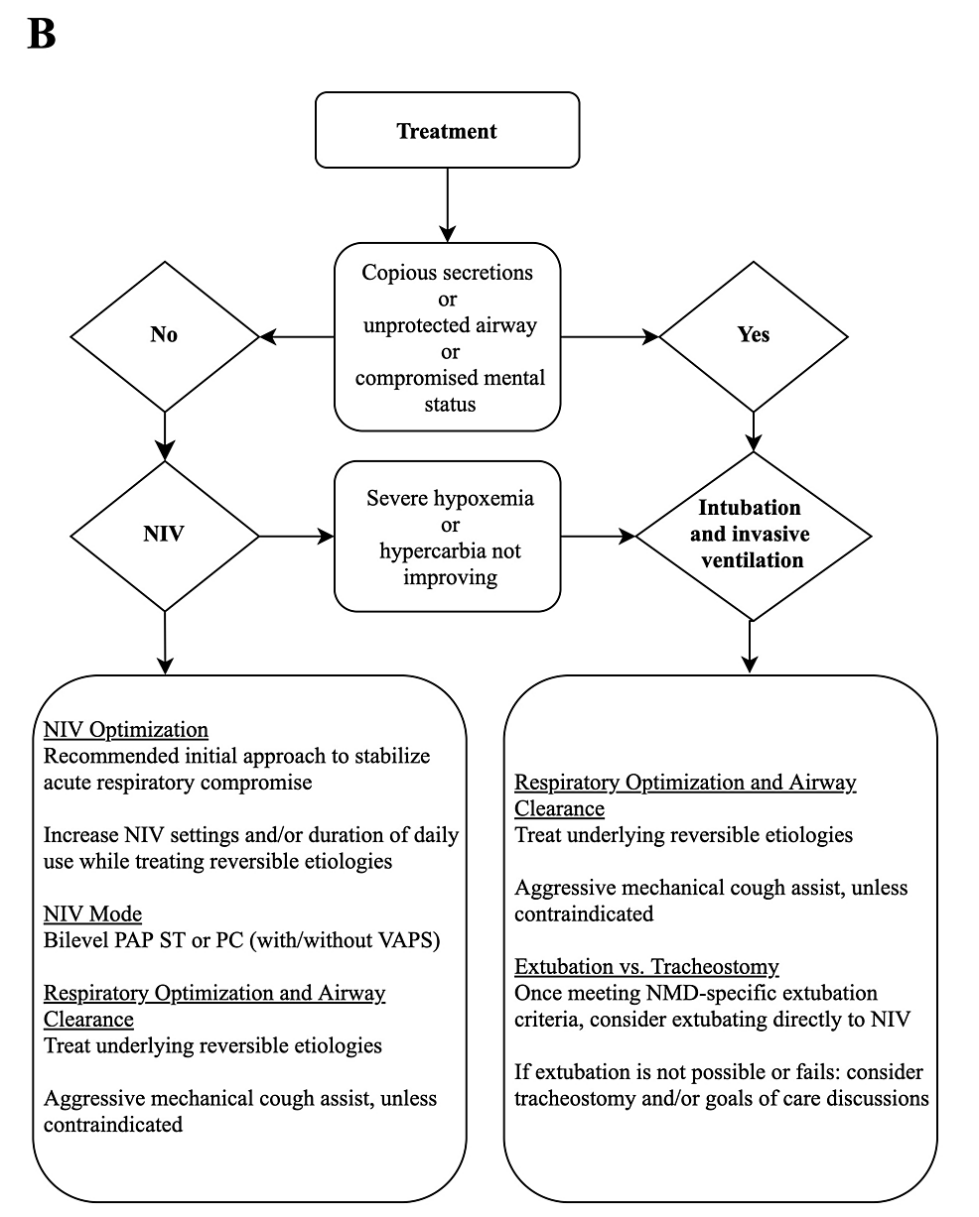
The critical care treatment of patients with acute on chronic neuromuscular failure B. Treatment defaults to noninvasive ventilation (NIV) options unless patient has contraindications or fails NIV trial, both NIV and invasive ventilation should be standardized in airway clearance. PAP: positive airway pressure; PC: pressure control; VAPS: volume-assured pressure support; NMD: neuromuscular disease.

In the setting of acute on chronic respiratory failure, NIV is most appropriate when alveolar hypoventilation is the driving factor, airway secretions are manageable, the airway is protected, and mental status is not compromised (Figure 2). If the patient is on home mechanical ventilation and acute respiratory failure can be stabilized on NIV, settings should be adjusted to optimize ventilation and oxygenation. Oxygen is usually required in conjunction with NIV in the acute setting. Supplemental oxygen without NIV however, is detrimental as it can decrease the hypoxic ventilatory drive, and worsening pulmonary function by two distinct mechanisms in a patient with preexisting impaired hypercapnic ventilatory drive and neuromuscular respiratory weakness. The first is via the Haldane effect, in which the oxygenation of hemoglobin shifts the serum bicarbonate buffer to CO_2_ production, worsening hypercapnea [7]. The second pathway involves worsened ventilation perfusion mismatch, by way of hyperoxia reversing hypoxic-induced vasoconstriction to poorly ventilated parts of the lung. 

Contraindications to NIV include significant cognitive impairment, unmanageable respiratory secretions, and severe hypoxemia and/or hypoventilation that cannot be reversed in a reasonable period of time with NIV. In patients on nocturnal-only mechanical ventilatory support at home, NIV may need to be implemented during the daytime and continued full time until the acute process is stabilized.

Of note, the duration of NIV dependence, including daytime or full time, was historically used as a metric to classify patients as “failing NIV” and hence as a decision point to initiate invasive mechanical ventilation. However, in patients with neuromuscular disease, NIV use is usually escalated to daytime first in order to prevent or stabilize acute hypercapnic respiratory failure. This escalation to daytime use should not be considered as a metric to define need for intubation unless respiratory failure is acute, rapidly progressive, and patient shows signs of clinical deterioration despite escalation of NIV to full time.

Finally, if the above NIV contraindications are present or if respiratory failure is rapidly progressive and hypoxemia and/or hypoventilation cannot be reversed safely, invasive mechanical ventilation should be pursued [8]. The most significant concern with invasive ventilation is the high likelihood of extubation failure due to underlying neuromuscular respiratory weakness. It is important to verify the patient’s wishes or advanced directives so that appropriate interventions can be implemented judiciously.

How to optimize ICU management

Multidisciplinary Care

At the time of ICU admission, multidisciplinary consultation and strategies from respiratory therapy, speech, and language therapy, and physical therapy should be initiated. High-quality standardized respiratory care is a primary driver of quality of life and is one of the few treatments shown to prolong survival and preserve respiratory strength in NMD. 

Noninvasive Mechanical Ventilatory Support

NMD patients with acute on chronic respiratory failure benefit from escalation of NIV support. Continuous positive airway pressure (CPAP) is contraindicated in NMD patients due to respiratory muscle weakness and the increased work of breathing that CPAP can place on the weak diaphragm during exhalation. Bi-level positive airway pressure (PAP) devices in spontaneous modes without back up respiratory rate (i.e. bilevel S) are not recommended either as the patient may often fail to trigger the device due to profound respiratory muscle weakness. Advanced modes of NIV with capability of providing back up respiratory rate and augmenting ventilation are advantageous as these may reduce rapid shallow breathing, improve gas exchange, allow respiratory muscle rest, and preserve respiratory muscle strength [9].

In general, high levels of pressure support are needed to optimally ventilate and address the respiratory muscle fatigue. Newer modes of NIV, such as volume-assured pressure support (VAPS) that guarantee a tidal volume and augment ventilation, are gaining popularity for ambulatory uses [10,11]. VAPS modality has theoretical advantages over set bilevel PAP as the VAPS algorithm provides more reliable goal tidal volume, improves minute ventilation, and is associated with decreased rapid shallow breathing index, although data are limited [11,12].

NIV Settings

NIV is recommended as the initial approach over invasive ventilatory support to stabilize acute respiratory compromise. We prefer pressure over volume regulated modes for noninvasive support. State-of-the-art reviews introduce the background and definitions for NIV modes and settings [10,13]. 

NIV options include basic bilevel PAP with back up respiratory rate to more advanced modes such as VAPS. Unlike bilevel PAP, in VAPS mode dynamic changes to pressure support are utilized to target or “guarantee” a tidal volume. Major settings for VAPS include tidal volume, maximum and minimum inspiratory positive airway pressure (IPAP max and IPAP min, respectively), expiratory positive airway pressure (EPAP), and back-up respiratory rate. We recommend a target tidal volume of 6-8 ml/kg (ideal body weight) for patients with otherwise normal lung compliance. Depending on the device manufacturer, pressure support maximum and minimum (pressure support max and min) are used instead of IPAP. Starting EPAP is recommended at 4-5 cm H_2_O to maintain upper airway patency. IPAP min should be about 5 cm H_2_0 above EPAP as a starting point for a naïve patient to NIV. IPAP max should be set high (10-20 cm H_2_O above IPAP min) to achieve adequate pressure support. If the IPAP max reaches high levels (greater than 25-30 cm H_2_O), then consultation with a NMD NIV specialist or intubation should be considered. Back-up respiratory rate should be set at 10-14 breaths per minute. For the majority of patients, knowing home ventilatory settings can be very helpful in adjusting IPAP and pressure support for initial set-up and when escalation of support is needed. A caveat to use of such home settings, however, is that many patients with acute worsening or as a result of the exacerbation require more support than that provided with baseline settings.

Minor NIV settings include rise time, inspiratory time (Ti), flow trigger sensitivity, and flow cycle sensitivity [10]. These settings are proprietary to device manufacturers and should be adjusted for patient comfort, synchrony, and status of respiratory compromise. Rise time is the time it takes for pressure to switch from EPAP to IPAP min, or the “pressurization” time. Depending on the device, it is usually set midrange (i.e. 3 or 300 msec) for neuromuscular patients. Ti is an important variable in neuromuscular respiratory weakness. Neuromuscular respiratory weakness can shorten the inspiratory cycle due to inability of diaphragm and inspiratory muscles to support full inspiration. This can result in tachypnea, shallow breathing and increased work of breathing if adequate tidal volume is not achieved. We recommend a higher Ti (1.0-1.2 second) to ensure full inspiratory cycle and adequate delivery of tidal volume per breath. Mandatory Ti is activated in pressure control mode on specific devices, while on others there is an option to program minimum Ti and maximum Ti. Lengthening the inspiratory cycle, however, is cautiously implemented in the acute setting and should not be done when patient is tachypneic, as it can lead to hyperinflation and ventilation perfusion mismatch. A high flow trigger sensitivity of 1-2 L/min is recommended as neuromuscular patients have difficulty triggering due to respiratory weakness. Use of traditional flow and trigger sensitivities are preferred over proprietary algorithms that aim to provide “auto-adaptive flow, trigger, and leak compensation”, specifically if they have not been studied in neuromuscular patients (e.g. Auto-Trak).

Use of well-fitted, full face mask interfaces (oronasal) is recommended to achieve adequate ventilation and minimize oral leak. If NIV is required during both day and nighttime, a nasal or pillows mask interface can be considered during daytime NIV for comfort, as it off-loads the constant pressure on the skin and may reduce the incidence of pressure ulcers. Then, at nighttime or while sleeping, many patients may switch to a full face mask. 

Invasive Mechanical Ventilation

Invasive mechanical ventilatory support is implemented when NIV fails. Invasive mechanical ventilation has advantages including full control of ventilation, easier access to clearing of respiratory secretions, and improved work of breathing. Main disadvantages include inability to extubate or extubation failure. If lung compliance is not an issue, we recommend use of either pressure or volume regulated modes with a target tidal volume of 6-8 ml/kg (ideal body weight). Synchronized intermittent mandatory ventilation (SIMV) or pressure support are not recommended as these modalities do not provide adequate respiratory support in spontaneous breaths. 

Extubation

Patients with NMD frequently fail spontaneous breathing or pressure support weaning trials due to underlying respiratory muscle weakness. Weaning parameters are also not helpful. Hence, unique NMD requirements should be met before extubation is considered, and these include: respiratory secretions are manageable with mechanical cough assist, SpO_2_ ≥ 94% without supplemental oxygen or that oxygen is being tapered off with FiO_2_ ≤ 40%, chest imaging showing improvement of acute pathology such as infiltrates, and patient is neurocognitively intact with stable cardiopulmonary status (Table 1). If these criteria are met, we recommend extubation directly to NIV utilizing high pressure support and back up respiratory rate. If available, continuous monitoring of end-tidal carbon dioxide (EtCO_2_) can be helpful during pressure support titration.

**Table 1:** Extubation protocol for intubated patients with neuromuscular respiratory failure Prior to extubation, neuromuscular disease (NMD)-specific criteria should be met, then most patients should be extubated directly to noninvasive ventilation (NIV). Prior to and after extubation, continue use of aggressive pharmacological and mechanical airway clearance. EtCO_2_: end tidal carbon dioxide; FiO2: fraction of inspired oxygen; IBW: ideal body weight; RT: respiratory therapist; SpO_2_: pulse oximetry; Tv: tidal volume.

Extubation Criteria	Post-Extubation NIV	Airway Clearance
SpO_2_ ≥ 94% without supplemental O_2_, however O_2 _is acceptable if oxygenation is consistently improving and FiO_2_ is ≤ 40%	Immediate initiation of NIV	Schedule mechanical cough assist every 2-4 hours for up to 12 hours after extubation
Do not follow weaning parameters, such as rapid shallow breathing index, as a marker for extubation candidacy	Use high pressure support and back up respiratory rate	Continue frequent pharmacological airway clearance with scheduled nebulizer therapy
Clear or improving chest imaging	Titration to achieve goal Tv, low respiratory rate, and SpO_2_ ≥ 94%	
Alert and following commands	Initiation of continuous EtCO_2 _monitoring	
Contingency plan in place if there is failure of extubation: goals of care clear for re-intubation, tracheostomy, or comfort care, appropriate airway equipment, RT at bedside, provider available for reintubation	Check blood gas 30-60 minutes after extubation to evaluate for hypercarbia and hypoxia	

Extubation of NMD patients often requires extended care at the bedside from a trained respiratory therapist. Scheduled cough assist therapy should be attempted every 2-4 hours for the first 12 hours post extubation. Peak cough expiratory flow >160 L/minute increases the likelihood that extubation will be successful at first attempt [14]. Frequent cough assist use at 30-50 cm H_2_O every 1-2 hours in intubated patients who were previously NIV-dependent and thought to be “unweanable” from invasive ventilation was successful in 99% of patients [15]. For treating SpO_2_ < 95%, considerations include increasing pressure support, evaluating for sources of unintentional leak within the circuit or mask interface, providing cough assist frequently to minimize atelectasis and clear respiratory secretions.

Airway Clearance

 Airway clearance of respiratory secretions by means of pharmacological agents and mechanical cough assist should be initiated and continued throughout the hospital stay. Patients with NMD are unable to effectively manage upper and lower airway secretions, which poses a key driver for extubation failure.

Respiratory Secretion Management 

Upper and lower respiratory secretions can be magnified in acute respiratory failure or pulmonary infection. Systemic anticholinergics are commonly used to minimize secretions, but these agents should only be used in a stable setting, as such ambulatory care. These agents should not be used when acute pulmonary infection (e.g. pneumonia) is present because of the risk of thickening secretions and impaired cough clearance. Scheduled nebulizer therapy with bronchodilators, anticholinergics, and hypertonic saline are recommended in the acute setting. Their frequency of use can be increased from home regimen. It is important to find a balance between drying up excessive secretions that require constant airway clearance and the equally problematic immobile thick secretions. 

Cough Augmentation

Patients with NMD are not able to mount a strong cough, and this is exacerbated with acute respiratory failure. Weak cough is associated with atelectasis, ineffective mucus clearance, and inability to wean from invasive ventilation. Characterizing cough strength and optimizing recruitment of remaining respiratory strength is thus essential. Effective cough strength is measured by peak cough flow (PCF), which is the maximum flow the patient can generate on expiration of a forceful cough. Normal adults have peak cough flows of 360 - 720 L/min. A value of < 270 L/min is ineffective and indicates initiation of manually or mechanically assisted cough, while a value of < 160 L/min signals low likelihood of successful extubation [16]. 

 At the time of ICU admission, mechanical cough augmentation, known as cough assist or mechanical insufflation-exsufflation, or “MIE” maneuvers should be implemented. MIE utilizes alternating rapid transitions of positive inspiratory pressure followed by negative expiratory pressure as a means for lung volume recruitment and assisted cough. Inspiratory and expiratory pressures depend on patient tolerance, but in general can be up to +50 and - 50 cm H_2_O, with 4 or 5 sets of breaths performed as often as needed to clear secretions and prevent atelectasis [17]. Finally, intrapulmonary percussive ventilation (IPV) can loosen lower airway secretions, but should be followed by MIE to bring up secretions. 

Use of MIE in NMD patients with respiratory tract infections significantly reduces treatment failure, as defined by the percentage requiring tracheostomy or invasive ventilation [18]. A systematic meta-analysis of prior randomized and semi-randomized trials on MIE found it to improve PCF compared to unassisted cough and to better clear mucus, but did not find sufficient evidence that MIE is superior to other cough augmentation techniques nor evidence on which to base clinical practice [19]. Outcomes, including survival, frequency of exacerbations, length of hospital stay and quality of life, are not well-described, although one study suggested that MIE may reduce cough-augmentation treatment time by 17 minutes when added to the standard airway clearance algorithm [20]. 

## Discussion

Critical care assessment and management of acute on chronic respiratory failure in a patient with NMD requires a multidisciplinary team combined with a well-defined protocol of broad diagnostics for assessing reversible and irreversible causes (i.e. the disease itself) of respiratory failure. Institutions benefit by pairing their personalized approach with literature-based treatment protocols, though the NMD population largely lacks data-drive recommendations. 

Standardizing an NMD-specific triage approach and treatment protocol facilitates delivery of optimal care for the two most common problems in this patient population: hypercapnic respiratory failure and ineffective airway clearance. The protocol described here addresses these challenges, and also presents a practical approach that may not yet be established in some hospital settings. The expert-derived best-practice approach reflected here is limited, however, primarily as a single-institution experience, yet it is based on literature-derived data [1-3-8,16]. Additional psychosocial and emotional factors also should be considered in this population; including conversations of intubation if a reversible etiology is not immediately identified in a patient who is uncertain of desire for invasive ventilation, and anxiety-driven hyperventilation 

Furthermore, the technical approach described provides a launchpad for (1) development of a provider's own approach and (2) future research investigations that can rigorously investigate this protocol versus others, especially between institutions and globally. Further opportunities exist for protocol and pathway development for intubated patients with NMD who require tracheostomy, as well as validation of respiratory therapy-driven NMD protocols and respiratory rehabilitation adapted to the severity of respiratory failure.

## Conclusions

Acute on chronic respiratory failure in patients with NMD requires critical care management and disease-specific approaches to mechanical ventilatory support, airway clearance, and extubation. A thorough understanding of the pathophysiology of neuromuscular respiratory weakness, identifying etiologies of acute respiratory failure, and experience in respiratory management including noninvasive positive pressure ventilation and mechanical airway clearance are critical to reversing the acute decompensation and a successful extubation.
